# Impact of Two Forms of Daily Preventive Zinc or Therapeutic Zinc Supplementation for Diarrhea on Hair Cortisol Concentrations Among Rural Laotian Children: A Randomized Controlled Trial

**DOI:** 10.3390/nu11010047

**Published:** 2018-12-27

**Authors:** Guy-Marino Hinnouho, Robin M. Bernstein, Maxwell A. Barffour, Charles D. Arnold, K. Ryan Wessells, Kethmany Ratsavong, Bangone Bounheuang, Sengchanh Kounnavong, Sonja Y. Hess

**Affiliations:** 1 Program in International and Community Nutrition, Department of Nutrition, University of California, Davis, CA 95616, USA; mabarffour@ucdavis.edu (M.A.B.); cdarnold@ucdavis.edu (C.D.A.); krwessells@ucdavis.edu (K.R.W.); syhess@ucdavis.edu (S.Y.H.); 2 Department of Anthropology, University of Colorado, Boulder, CO 80309, USA; robin.bernstein@colorado.edu; 3 Health and Society Program, Institute for Behavioral Science, University of Colorado, Boulder, CO 80309, USA; 4 Public Health Program, College of Health and Human Services, Missouri State University, Springfield, MO 65897, USA; 5 Lao Tropical and Public Health Institute, Ban Kaognot, Sisattanack District, Vientiane 01030, Laos; kethmany.ratsavong@gmail.com (K.R.); bangonbounheuang@gmail.com (B.B.); sengchanhkounnavong@hotmail.com (S.K.)

**Keywords:** zinc supplementation, micronutrient powder, MNP, hair cortisol, chronic stress, young children, Lao PDR

## Abstract

Zinc supplementation has been shown to reduce the morbidity burden among young children, and may reduce chronic stress. Hair cortisol has been promoted as an indicator of chronic stress. We assessed the impact of different strategies for delivering supplementary zinc on hair cortisol concentrations (HCC) in young Laotian children and examined risk factors associated with HCC. In a randomized double-blind controlled trial (NCT02428647), children aged 6–23 mo were randomized to one of four intervention groups and followed for ~36 weeks: daily preventive zinc (PZ) tablets (7 mg/day), daily multiple micronutrient powder (MNP) sachets (containing 10 mg zinc and 14 other micronutrients), therapeutic zinc (TZ) supplements for diarrhea treatment (20 mg/day for 10 days) or daily placebo powder. HCC of 512 children was assessed at baseline and endline. ANCOVA and linear regression models were used to assess group differences in HCC and to examine the risk factors associated with HCC, respectively. At enrollment, mean HCC was 28.8 ± 43.9 pg/mg. In models adjusted for age at enrollment, health district, and baseline HCC there was no overall effect of the interventions on endline HCC and change in HCC. When controlling for additional predetermined covariates, there was a marginally significant effect on change in HCC (*p* = 0.075) with a slightly lower reduction of HCC in TZ compared to PZ (mean change (95% CI): −4.6 (−7.0; −2.3) vs. −9.4 (−11.7; −7.0) pg/mg; *p* = 0.053). At baseline, consumption of iron rich foods was negatively associated with HCC, whereas AGP (α1-acid glycoprotein) levels, elevated AGP and C-reactive protein and high soluble transferrin receptor were positively associated with HCC. In young Laotian children, MNP, PZ and TZ had no impact on HCC. The marginal difference in change in HCC between the PZ and TZ groups was too small to be considered of health significance.

## 1. Introduction

Chronic stress, as a result of economic deprivation, inadequate nutrition and/or poor health status, experienced by children in low-income countries, can have detrimental consequences on their wellbeing, physical and mental health later in life [1,2,3]. Although unclear, the mechanism underlying the relationship between chronic stress and adverse health outcomes is hypothesized to be an increased activation of the hypothalamic–pituitary–adrenal (HPA) axis [4], which may trigger an elevation in circulating cortisol levels [5].

Traditional biological specimens for cortisol measurement include saliva, blood or urine samples, each with its advantages and disadvantages [6]. Serum and saliva are subject to major physiological daily fluctuations and thus reflect acute stress [7], limiting their usefulness as indicators of stress levels over long periods of time [8]. In addition, blood collection for assessment of serum cortisol is invasive and stressful for children and may increase circulating cortisol levels, while collection of saliva, although less-invasive, presents other challenges related to protocol compliance [9] and collection methods [10]. In contrast to serum and saliva samples, a 24-h urine collection is non-invasive and is used for measuring cortisol secretion over a prolonged period [11]. However, a 24-h urine collection is time-consuming, laborious and presents some difficulties in regards to the analytical method and protocol compliance [8].

There is growing evidence that hair offers an alternative suitable matrix for monitoring long-term cortisol profiles by capturing systemic cortisol exposure over longer periods of time [8,12,13]. Hair is easy to collect, non-invasive, less stressful and unlike blood, saliva and urine which require frozen storage, can be stored at room temperature in a sealed envelope. Only a small amount of hair is required to assess information prior to hair collection [13] and analysis of sections along the hair shaft enables differentiation between several time periods in the past [6]. Furthermore, it has been reported that hair cortisol is stable over time [14], has a high overall validity and test-retest reliability [15] and is not found to be influenced by acute stress [16].

While hair cortisol concentration (HCC) has been widely used as a biomarker of chronic stress in studies among adults [17,18], it is only emerging now as a promising marker of childhood chronic stress. To date there is no consensus on the best approach to assess chronic stress in young children [19]. The main focus of the existing literature on HCC as a biomarker of chronic stress in young children has been limited to exploring the main determinants of HCC in terms of chronic stress exposure, such as low socio-economic status and maternal distress [20]. Here, we investigate the utility of HCC as a stress biomarker in the context of a randomized controlled trial of zinc supplementation among 6 to 23 months old children.

Adequate zinc nutrition is essential for human health because zinc is involved in numerous metabolic processes as a catalyst, a regulatory ion or structural element of proteins [21]. Because zinc participates in so many metabolic pathways, zinc deficiency affects multiple physiological systems, children’s physical growth, the risk and severity of a variety of infections and pregnancy outcomes. Preventive zinc supplementation provided daily in form of tablets or syrup has been shown to reduce the incidence of diarrhea and acute lower respiratory infection among young children [22,23,24,25] which in turn may reduce chronic stress. Zinc is also commonly included in multiple micronutrient powders (MNP), which have consistently been found to reduce the risk of anemia and increase iron status [26]. However, a recent meta-analysis of MNPs found a significant association between MNP and an increase in diarrhea incidence [27], which was assumed to be due to potentially adverse effects of iron. Lastly, therapeutic zinc supplementation, as recommended by the World Health Organization (WHO) and UNICEF [28], shortens the duration of diarrhea and reduces the number of children whose diarrhea persists for 7 days [29].

Hair zinc, a biological marker of long-term zinc nutrition, has been reported to be negatively associated with HCC among 4–6 years old children in Vancouver [30], and the authors speculated that the inverse relationship between zinc and cortisol concentrations in hair may suggest some chronic stress in the study population. Whether the above-mentioned various regimens of zinc supplementation with or without other micronutrients have an impact on HCC is unknown. Therefore, in the present study, we aimed to: (1) assess the impact of different strategies for delivering supplementary zinc on HCC in young Laotian children, (2) examine the risk factors associated with baseline HCC and, (3) assess the association between endline HCC and the diarrhea and fever burden in the previous 3 months.

## 2. Materials and Methods

### 2.1. Ethical Approval

Ethical approval of the trial was provided by the National Ethics Committee for Health Research (Lao PDR) and the Institutional Review Board of the University of California, Davis (UC Davis). The trial was registered at www.clinicaltrials.gov (NCT02428647). 

### 2.2. Study Design

We implemented a randomized controlled double-blind community-based trial, known as the Lao Zinc Study, from September 2015 until April 2017 in rural communities in Khammouane Province, central Lao PDR with the aim to compare two forms of daily preventive zinc supplementation (tablets and MNP) versus therapeutic zinc supplementation for diarrhea on young children’s physical growth and other health outcomes. A detailed protocol of the Lao Zinc Study has been published elsewhere [31]. Briefly, written informed consent (documented by either a signature or a fingerprint in the presence of a neutral witness) was obtained from one of the child’s primary caregivers (mother, father or legal guardian). Children were considered eligible if they were 6–23 months of age, and their families accepted weekly visits, planned residency within the study area for the duration of the study and signed informed consent. Children were ineligible if they met one of the following criteria: severe anemia (Hb < 70 g/L), weight-for-length *z*-score (WLZ) < −3 [32], presence of bipedal edema, severe illness warranting hospital referral, congenital abnormalities potentially interfering with growth, chronic medical condition (e.g., malignancy) requiring frequent medical attention, known human immunodeficiency virus (HIV) infection of index child or child’s mother, currently consuming zinc supplements or current participation in another clinical trial.

### 2.3. Sample Size Estimation 

A sample size of 133 children per study intervention group was estimated to be able to detect a difference in mean HCC between any two intervention groups with an effect size of 0.4, a power of 80% and a type I error of 5%. Considering the absence of information of the impact of micronutrient interventions on HCC, the effect size of 0.4 was informed by other indicators of micronutrient status such as plasma zinc and ferritin [22,33]. An attrition rate of 30% was considered to account for drop out and possible failure to successfully collect hair at both time points (total sample size = 760). Sample size estimation was done with the use of SAS software (version 9.4; SAS Institute, Inc., Cary, NC, USA).

### 2.4. Randomization and Intervention Products

Eligible children were individually randomized to one of the four following intervention groups: (1) the preventive zinc (PZ) group, receiving 7 mg of a daily preventive dispersible zinc supplement plus a placebo therapeutic tablet for diarrhea; (2) the MNP group receiving a daily preventive MNP containing 10 mg zinc as zinc gluconate and 6 mg iron as ferrous fumarate along with 13 other micronutrients plus a placebo therapeutic tablet for diarrhea; (3) the therapeutic zinc (TZ) group, receiving a daily preventive placebo tablet plus 20 mg of therapeutic zinc for diarrhea for 10 days; or (4) the control group, receiving a daily placebo preventive powder plus a therapeutic placebo tablet for diarrhea. This new MNP formulation containing a lower amount of iron and a higher amount of zinc dose than current formulations was tested to address the concerns of potential adverse effects of MNP on diarrhea. In addition to zinc and iron, it contained 0.56 mg copper as copper sulphate anhydrous, 17 μg selenium as selenium selenite, 90 μg iodine as potassium iodate, 400 μg RE vitamin A, 5 μg vitamin D (cholecalciferol), 5 mg vitamin E (dl-α-tocopherol acetate), 30 mg ascorbic acid, 0.5 mg thiamin, 0.5 mg riboflavin, 6 mg niacin, 0.5 mg vitamin B-6, 0.9 μg vitamin B-12, and 150 μg folic acid. All children received oral rehydration salts (ORS) to be taken during diarrhea episodes.

The preventive zinc, therapeutic zinc and placebo tablets were produced by Nutriset SAS (Malaunay, France). The powder supplements (MNP and placebo) were produced by DSM Fortitech Asia Pacific Sdn Bhd (Banting, Malaysia). Caregivers were instructed to dissolve the tablet supplements (one dose per day for PZ; one tablet daily for 10 days as part of diarrhea management for TZ) with clean water or breast milk and spoon feed the child 30 min before or after a meal. For the powder supplements, they were advised to mix the entire content of the sachet with a semi-solid or mashed food.

### 2.5. Data Collection

Children’s anthropometry (weight, length and mid-upper arm circumference (MUAC)) were measured at baseline and endline and maternal weight and height were measured once. All anthropometric measurements were completed in duplicate [34]. Information on maternal and household demographic and socio-economic status (education, occupation, ethnicity, household size and composition, housing material, household assets, and land ownership), food security and hygiene and sanitation practices were collected at baseline. Information on infant and young child feeding (IYCF) practices (breastfeeding, formula feeding, 24-h and 7-day food frequency questionnaire) were collected at baseline and every four weeks.

At baseline and endline, venous blood samples were collected to assess biomarkers of nutritional (ferritin, soluble transferrin receptor (sTfR), retinol binding protein (RBP)) and inflammatory (C-reactive protein (CRP) and α1-acid glycoprotein (AGP)) status by combined sandwich enzyme-linked immunosorbent assay (ELISA) technique at VitMin Lab (Willstaett, Germany) [35].

### 2.6. Morbidity Surveillance and Supplement Administration

Children enrolled in the trial remained under observation and received their assigned supplements daily for a period of ~36 weeks. Each household was visited weekly by a morbidity surveillance worker who recorded reported morbidity symptoms for each day of the previous week and delivered the respective preventive supplements. Recorded morbidity symptoms included fever, diarrhea (number and consistency of stools), respiratory symptoms (cough and nasal discharge) and any other symptoms of concern. Axillary temperature was measured once every four weeks and whenever fever was reported within 24 h of the home visit.

### 2.7. Hair Samples Collection and Hair Cortisol Analyses

Due to the large study area causing logistical challenges, participants were selected for separate subgroup analyses based on logistical feasibility [31]. In an effort to obtain information on micronutrient and inflammatory status and HCC, venous and hair samples were collected in the same subgroup of children from a convenience sample. A total of 788 children provided hair samples at baseline and 694 children at endline, of which 529 children who had both baseline and endline samples were selected for analyses of HCC. The endline hair samples were collected ~32–36 weeks after the baseline hair collection. Approximately 10–20 mg of hair was cut as close to the scalp as possible from the center of the nape of the neck (~2 cm above hair line), or a nearby location, in case of insufficient hair. All hair samples were placed in small paper envelopes and subsequently stored in individual Ziploc bags, at room temperature. 

Hair cortisol analysis was successfully performed in 512 children who provided adequate hair quantity at both baseline and endline assessments. Approximately 10 mg of hair was weighed (mean hair weight ± SD was 10.06 ± 0.75 mg) and placed into 2 mL polypropylene tubes. Hair was washed with isopropanol, dried under a stream of air in a fume hood for 48 h, and then ground with one stainless steel ball per tube for 10 min at 25 mHz, using a Retsch 400MM ball mill (Verder Scientific, Newton, PA, USA). Ground samples were incubated with 1 mL of HPLC-grade methanol on a rotating platform overnight at 170× *g*. The next day, samples were centrifuged for 12 min at 4200× *g*, and 875 µL of supernatant was removed and transferred to a new 2 mL tube. The supernatant was dried down under a constant stream of nitrogen gas for ~15 min using a Micro-Vap system (Organomation, Berlin, MA, USA). Samples were then reconstituted with 0.5 mL EIA buffer solution, and reconstituted samples were assayed using a commercially available salivary cortisol kit (Salimetrics, State College, PA, USA) previously validated for use with human hair samples [30].

### 2.8. Definitions

Stunting, underweight and wasting were defined as length-for-age *z*-scores (LAZ) < −2, weight-for-age *z*-scores (WAZ) < −2 and weight-for-length *z*-scores (WLZ) < −2 respectively [32]. Low MUAC was defined as MUAC ≤ 12.5 cm. Elevated CRP and AGP were defined as CRP > 5 mg/L and AGP > 1 g/L, respectively. Low ferritin was defined as plasma ferritin (pF) < 12 µg/L and high sTfR as sTfR > 8.3 mg/L. A child was considered breastfed if breastfeeding was reported at least once in the past month.

### 2.9. Statistical Analyses

A statistical analyses plan describing the statistical procedures was published prior to the analyses [36]. Analyses were done based on complete-case intention-to-treat, and the intervention group was considered the primary exposure variable. All analyses were undertaken using Stata 14 (StataCorp 2015, College Station, TX, USA).

To assess the first objective, we examined the difference in mean HCC at endline and the magnitude of change in HCC between baseline and endline among the four intervention groups. Baseline and endline HCC were log-transformed to normality but the difference in baseline and endline was normally distributed and thus not transformed. ANCOVA regression models were used to assess treatment effect on HCC in minimally adjusted models (including baseline value of HCC, age at baseline and health district) and fully-adjusted models (including variables in the minimally adjusted models and pre-specified variables determined to be associated with outcome (*p* < 0.1)). Potential effect modifiers by baseline variables were explored by incorporating interaction terms in the statistical models. Interaction terms were further examined if marginally significant (*p* < 0.1). 

For the second objective, we examined potential risk factors associated with HCC at baseline. Linear regression models were used to assess these associations and analyses were adjusted for age at baseline, sex and health district. Principal components analysis was applied to available indicators of household socio-economic status, education, income, ownership of lands and hygiene and sanitation practices to derive a SES index [37]. Food security was defined using the household food insecurity access scale (HFIAS) [38] and information on IYCF practices (breastfeeding, dietary diversity and food frequency) were used to estimate the adequate dietary diversity (ADD), minimum meal frequency (MMF) and consumption of iron-rich foods as specified by WHO [39,40].

For the third objective, we explored associations between endline HCC and morbidity burden between mid-point (18 weeks after enrollment) and endline corresponding to the previous 3–4 months. This time period was chosen because a previous study reported that a hair sample of 2–3 cm reflects the average HCC over the previous 2–3 months, assuming a hair growth rate of approximately 1 cm/month [17]. These associations were examined using linear regression models adjusted for intervention groups, age at baseline, sex, and health district. Morbidity outcomes assessed were the longitudinal incidence of diarrhea and reported and measured fever. These outcomes were defined as a dichotomous (yes/no) variable based on the occurrence of at least one episode (yes) during the selected time frame. 

## 3. Results

### 3.1. Study Population

**Figure 1:**
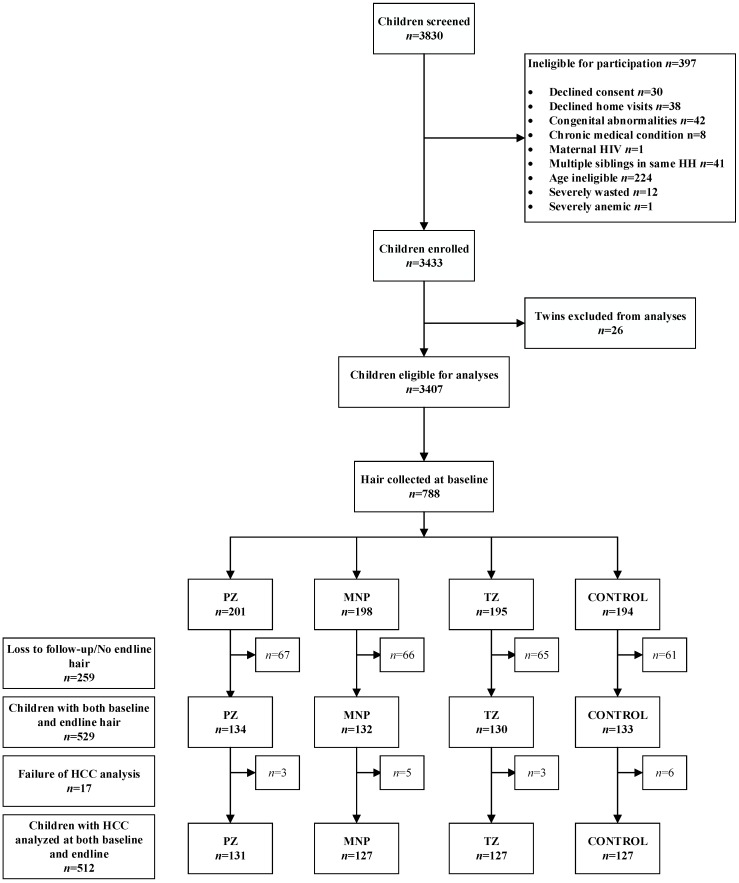
Lao Zinc study flow diagram for hair collection. MNP = Micronutrient powder; PZ = preventive zinc; TZ = Therapeutic zinc.

From a total of 529 children who provided hair samples at both baseline and endline assessments, HCC was successfully assessed in 512 children (*n* = 131 in the PZ group and *n* = 127 in the other 3 groups, respectively; Figure 1).

Mean age at baseline was 15.6 ± 5.0 months (Table 1). The prevalence of stunting and underweight was high at 38.2% and 27.2%, respectively but wasting was less common (6.3%). The majority of children were breastfed (64.4%) but only about one third of the children (35.9%) had adequate dietary diversity at baseline. A total of 16.8% of the households reported to be severely food insecure. CRP and AGP concentrations were elevated in 11.3% and 20.0% of children respectively. Mean HCC at baseline was 28.8 pg/mg (median (IQR): 21.7 (13.8–35.1)).

Compared to the children participating in the main trial, those included in this analysis were older (15.6 mo vs. 14.0 mo, *p* < 0.001), more likely to be female (55.3% vs. 48.4%, *p* = 0.004), and severely food insecure (16.8% vs. 12.4%, *p* < 0.001). They were less likely to be breastfed (64.4% vs. 74.4%, *p* < 0.001) but have better reported IYCF practices (adequate dietary diversity: 35.9% vs. 10.1%, minimum meal frequency, 55.7% vs. 46.9%, consumption of iron-rich foods: 79.9% vs. 64.7%; all *p* < 0.001). The prevalence of stunting, wasting and underweight were not significantly different between the 2 cohorts of children (data not shown).

### 3.2. Impact of the Study Interventions on HCC 

In both minimally adjusted (adjusted for baseline value of HCC, age, district) and fully adjusted (adjusted for baseline value of HCC, age, district, sex, LAZ, WAZ, wasting, health center and maternal marital status) models, endline HCC did not differ significantly across the four groups (*p* = 0.231 and *p* = 0.260, respectively; Table 2). There was no overall effect on change in HCC (*p* = 0.121) in the minimally adjusted model. There was a statistically marginal effect on change in HCC in the fully adjusted model (*p* = 0.075). Specifically, the reduction in HCC was slightly lower in the TZ group than in the PZ group (mean change (95% CI): −4.6 (−7.0; −2.3) vs. −9.4 (−11.7; −7.0) pg/mg, respectively; *p* = 0.053). 

### 3.3. Risk Factors Associated with HCC at Baseline

In analyses examining potential risk factors associated with HCC at baseline (Table 3), maternal age, education, marital status and BMI were not associated with baseline HCC. Similarly, SES index and HFIAS were not associated with HCC at baseline. However, baseline child consumption of iron rich foods was negatively associated with HCC at baseline, whereas AGP concentration, elevated AGP, elevated CRP and high sTfR were positively associated with HCC at baseline. In addition, the associations between baseline HCC and breastfeeding, child weight, length, low MUAC and CRP concentration at baseline were marginally significant (*p* < 0.1).

### 3.4. Association Between Endline HCC and Diarrhea and Fever in the Previous 3 Months

Caregivers reported at least one episode of diarrhea in 46.5% of children, while fever was reported or measured at least once among 74.6% and 11.7% of children, respectively. Neither the presence of reported diarrhea (*p* = 0.666), nor reported (*p* = 0.520) or measured (*p* = 0.560) fever in the previous 3–4 months were associated with HCC at endline (Table 4).

## 4. Discussion

Growing evidence indicates that HCC may be an objective biomarker of chronic stress, and given that zinc supplementation has been reported to reduce the morbidity burden, we hypothesized that zinc supplementation may reduce chronic stress by affecting the immune function and the morbidity burden. Thus, we examined the impact of different strategies for delivering supplementary zinc on HCC among young Laotian children. In our study population of 512 young children aged 6–23 months at enrolment, we found no significant impact of the different zinc supplementation strategies on HCC. However, there was a marginal difference in change in HCC between the preventive zinc and therapeutic zinc groups after statistical adjustment. This difference was too small to be considered of health significance. 

To explore whether HCC may be a useful indicator of nutritional and health status during early childhood, we examined the association between HCC and potential risk factors and found that children’s CRP, AGP concentrations, high sTfR levels and lack of consumption of iron rich foods were risk factors for elevated HCC at baseline. We are uncertain why or how breastfeeding would be inversely associated with HCC and assume that this may be a chance finding. In contrast, the child’s stunting and wasting status were not associated with baseline HCC. We also found no associations between maternal characteristics (age, education, BMI), household SES status and baseline HCC. Furthermore, we evaluated the association between HCC and longitudinal morbidity and found that diarrheal and febrile morbidity in the previous 3–4 months did not translate into an increased HCC at endline.

It is unclear whether our intervention had no impact on HCC because (1) the tested interventions had no impact on chronic stress, or (2) the study population was not under chronic stress or (3) HCC is not a sensitive marker of chronic stress in this population. We will address each of these points below. 

As previously reported, the present study found no overall impact of PZ, MNP and TZ on growth and morbidity outcomes, such as diarrhea, fever and respiratory distress [41,42], although TZ reduced the incidence and duration of diarrhea episodes in older children (>18 mo), but not in younger ones. This beneficial impact on diarrhea outcomes did not result in lower HCC in the TZ group, nor did age have a modifying effect on HCC. Although MNP had no overall impact on growth and morbidity, we found that the provision of MNP was associated with a small adverse effect on linear growth among non-anemic children and on diarrhea among children with inherited hemoglobin disorders [43]. Neither the beneficial nor the adverse effects observed in the Lao Zinc Study were reflected in the HCC, which was possibly due to the small magnitude of the effect or the limited sample size of children with HCC results in the respective subgroups. Another reason for the lack of impact of our interventions could be the relatively short duration of the intervention. We followed our study participants for ~36 weeks. Although this duration is consistent with previous studies of preventive zinc supplementation that found an impact on growth and morbidity outcomes [26,44,45], the duration may have been inadequate to affect an indicator of chronic stress such as HCC. To the best of our knowledge, this study is the first intervention study to assess the impact of different strategies for delivering supplementary zinc or other micronutrients on HCC, making any comparison with the existing literature difficult. More evidence from supplementation trials is needed to understand whether zinc and MNP supplementation has an impact on chronic stress as reflected in HCC. 

Another reason for the apparent lack of impact of our interventions on HCC may be that our study population was not under chronic stress, and thus HCC would not respond to supplementation. There are currently no cut-offs for HCC to define chronic stress. Mean baseline HCC in our study population was 28.8pg/mg which is similar to mean HCC (27.33 pg/mg) in 12 month old infants in Boston [46] but higher than the mean HCC of the majority of the previous studies [20], although there is a wide range in HCC reported in the literature, ranging from 5.0 pg/mg in 4–5 years old Dutch children [47] to 40.9 pg/mg in 1-9 years old German children [48] and 535.3 pg/mg in 3–18 years old subjects from Central African Republic and Ethiopia [49]. Unfortunately, there are no published studies on HCC in 0–2 years old infants in low- and lower-middle-income countries. There was a high prevalence of stunting (38.2%), underweight (27.2%), zinc deficiency (75.4%) and iron deficiency (26.1%) in the study population, suggesting that many of these children may experience other types of chronic stress. 

Even though studies have used direct validation and different strategies to indirectly validate hair cortisol as a biomarker of chronic stress [50], only weak correlations have been reported between psychological tests (perceived stress for example) and HCC [51,52], raising some concerns regarding its utility and applicability as a sensitive marker of childhood chronic stress. The risk factors associated with HCC in children have been previously examined [20] and mixed findings have been reported in regards to household socio-economic status [30,53,54], ethnicity [30,55], maternal education [30,56], child age [47,48] and gender [48,57]. It is worth mentioning that studies which reported socio-economic status, maternal education and child age to be risk factors of HCC [30,53] included older children than those in the present study. Child age was not an effect modifier in our study. However, the age range in the present study was limited compared to some of the prior investigations in other populations mentioned above. Although the present study was implemented in rural communities of central Laos, children included in our study were young (6–23 mo) and may have either not had enough cumulative exposure to the effects of low socioeconomic status, or may use effective buffering mechanisms such that these associations do not emerge. 

The association between HCC and child nutritional and health status has not previously been examined. We found that HCC was not significantly associated with child anthropometric measurements such as length, weight, MUAC, LAZ, WAZ, WLZ, stunting, wasting and underweight. In addition, no previous study has examined the associations between HCC and morbidity and we found no association between HCC and morbidity in the 3–4 months prior to endline hair collection. Moreover, HCC was neither associated with factors such as food security, adequate dietary diversity and minimum meal frequency. This lack of association between HCC and child nutritional and health status questions and challenges the usefulness of HCC as a sensitive marker of child nutritional and health status.

Previous studies found a high variability in HCC among different age groups [20]. Hair sample collection method is unlikely to be a source of variability between our findings and other studies. As in other studies [58,59], we cut hair in accordance with the guidelines published by the Society of Hair Testing [60] from above the nape of the neck as close to the scalp as possible. Hair from this region has been reported to be the standard for hair analysis and to have higher cortisol levels compared with hair from other regions [61]. However, cortisol in hair is usually quantified using enzyme linked immunosorbent assay (ELISA) and high performance liquid chromatography-mass spectrometry (HPLC/MS) [62] and given the multitude of commercially available cortisol kits, the HCC detection method is more likely to be a source of the variability found. Other potential biases of the study could include the color of hair, but considering that the study was implemented in Laos, there was very little diversity in hair color. Data on hair care characteristics in terms of washing frequency, and use of hair products or type of hair products were not collected mainly because of the age-range of our study participants (6–32 months old). Previous studies that collected data on hair care characteristics were done in adults [63] or in older children (4–14 years old) [47] and findings from these studies have reported that HCC were not affected by hair color, hair washing frequency or use of hair products. 

A notable strength of this study is its randomized placebo-controlled trial design, the high participation rate and the rigorous data collection, which included both regular training of data collectors and frequent supervision. The vast amount of collected data allowed the investigation of potential risk factors associated with HCC. This study also has some limitations. Adherence to the supplementation was based on weekly caregiver reports, which has been shown to be unreliable [64]. However, plasma zinc concentrations increased in the PZ and the MNP groups and ferritin concentrations increased in MNP group as expected and thus suggest that adherence was adequate [41]. In addition, hair samples were collected from a convenience sample for logistical reasons. However, hair collection was implemented among all four intervention groups simultaneously until the respective target sample size was achieved [31].

## 5. Conclusions

In this population of young Laotian children, supplementation with zinc alone or combined with other micronutrients as an MNP had no impact on HCC. The marginal difference in change in HCC between preventive and therapeutic zinc groups was too small to be considered of health significance. Additional research is needed to examine the usefulness of HCC as an indicator of child health status.

## Figures and Tables

**Table 1:** Child, maternal and household characteristics of the study participants at baseline by intervention group ^1^.

Characteristics	All (*n* = 512)	PZ (*n* = 131)	MNP (*n* = 127)	TZ (*n* = 127)	CONTROL (*n* = 127)
Age, mo	15.6 ± 5.0	15.3 ± 5.2	15.9 ± 5.0	15.8 ± 4.8	15.4 ± 5.0
Gender, female	283 (55.3)	79 (60.3)	64 (50.4)	73 (57.5)	67 (52.8)
Breastfeeding	298 (64.4)	72 (63.7)	70 (62.5)	78 (65.6)	78 (65.6)
^2^ Adequate dietary diversity	167 (35.9)	39 (34.5)	39 (34.5)	38 (31.7)	51 (42.9)
^3^ Minimum meal frequency	258 (55.7)	63 (55.8)	60 (53.6)	65 (54.6)	70 (58.8)
Consumption of iron rich foods	371 (79.8)	83 (73.5)	93 (82.3)	101 (84.2)	94 (79.0)
Child anthropometric measures					
Length, cm	73.8 ± 5.4	73.3 ± 5.6	74.0 ± 5.2	73.6 ± 5.2	74.5 ± 5.6
Weight, kg	8.5 ± 1.3	8.3 ± 1.3	8.5 ± 1.3	8.5 ± 1.3	8.8 ± 1.3
MUAC, cm	13.9 ± 1.0	13.8 ± 1.0	13.9 ± 1.0	14.0 ± 1.0	14.1 ± 1.0
LAZ	−0.71 ± 1.08	−1.79 ± 1.10	−1.79 ± 1.10	−1.86 ± 1.07	−1.41 ± 1.07
WAZ	−1.42 ± 0.99	−1.52 ± 1.00	−1.52 ± 1.04	−1.51 ± 0.96	−1.14 ± 0.93
WLZ	−0.74 ± 0.86	−0.81 ± 0.89	−0.82 ± 0.86	−0.76 ± 0.87	−0.57 ± 0.82
Stunting	195 (38.2)	52 (40.0)	52 (40.9)	54 (42.5)	37 (29.1)
Wasting	32 (6.3)	11 (8.5)	12 (9.5)	7 (5.5)	2 (1.6)
Underweight	139 (27.2)	40 (30.8)	41 (32.3)	38 (29.9)	20 (15.8)
Elevated CRP (>5 mg/L)	55 (11.3)	16 (12.6)	9 (7.44)	14 (11.5)	16 (13.9)
Elevated AGP (>1 g/L)	97 (20.0)	27 (21.3)	24 (19.8)	22 (18.0)	24 (20.9)
Hair cortisol concentrations, pg/mg	21.7 (13.8–35.1)	27.0 (14.7–38.3)	19.4 (12.5–29.7)	21.0 (13.4–34.4)	21.5 (14.0–34.0)
Maternal education, primary or lower	267 (53.3)	72 (56.7)	67 (52.8)	64 (51.6)	64 (52.0)
Maternal BMI, kg/m^2^	22.0 ± 3.3	21.8 ± 3.1	22.2 ± 3.2	21.9 ± 3.4	22.0 ± 3.5
HFIAS					
Food secure	131 (25.9)	35 (27.3)	35 (28.2)	25 (19.7)	36 (28.6)
Mildly food insecure	148 (29.3)	35 (27.3)	37 (29.8)	39 (30.7)	37 (29.4)
Moderately food insecure	141 (27.9)	26 (20.3)	36 (29.0)	42 (33.1)	37 (29.4)
Severely food insecure	85 (16.8)	32 (25.0)	16 (12.9)	21 (16.5)	16 (12.7)

^1^ Values presented as n (%), means ± SDs or medians (IQRs). AGP: α_1_-acid glycoprotein; BMI: body mass index; CRP: C-reactive protein; HFIAS: household food insecurity access scale [38]; LAZ: length-for-age *z*-score; MNP: micronutrient powder; MUAC: mid-upper arm circumference; PZ: preventive zinc; TZ: therapeutic zinc; WAZ, weight-for-age *z*-score; WLZ, weight-for-length *z*-score. ^2^ Adequate dietary diversity: Proportion of children 6–23 months of age who receive foods from 4 or more food groups. ^3^ Minimum meal frequency: Proportion of breastfed and non-breastfed children 6–23 months of age who receive solid, semi-solid, or soft foods the minimum number of times or more.

**Table 2:** Effects of daily preventive zinc, micronutrient powder or therapeutic zinc for diarrhea on hair cortisol concentrations (HCC) among Laotian children ^1^.

	*n*	PZ	MNP	TZ	CONTROL	*P*
**^2^ HCC at endline, pg/mg**						
Minimally adjusted model	512	14.3 (12.5; 16.4)	13.3 (11.6; 15.3)	16.1 (14.0; 18.4)	13.6 (11.8; 15.6)	0.231
Fully adjusted model	499	13.6 (11.8; 15.6)	13.1 (11.4; 15.1)	15.9 (13.8; 18.3)	13.7 (11.9; 15.9)	0.260
**^3^ Change in HCC, pg/mg**						
Minimally adjusted model	512	−8.9 (−11.2; −6.6)	−7.8 (−10.2; −5.4)	−5.0 (−7.4; −2.6)	−7.8 (−10.2; −5.4)	0.121
Fully adjusted model	499	−9.4 (−11.7; −7.0)	−7.7 (−10.1; −5.4)	−4.6 (−7.0; −2.3)	−7.2 (−9.7; −4.8)	0.078

^1^ Estimates are means (95% CI). ANCOVA regression models were used to examine the difference in mean HCC at endline and the magnitude of change in HCC. Minimally adjusted: adjusted for baseline value of HCC, age, and district. Fully adjusted: Minimally adjusted + sex, LAZ, WAZ, wasting, health center and maternal marital status. HCC: Hair cortisol concentrations; MNP: micronutrient powder; PZ: preventive zinc; TZ: therapeutic zinc. ^2^ Results shown as geometric mean (95% CI) (HCC were log-transformed and then the estimates were back transformed using excel’s exponential function). ^3^ Results shown as arithmetic mean (95% CI) and change is from baseline to endline.

**Table 3:** Associations between baseline HCC and potential risk factors among Laotian children ^1^.

Variables	Percent Change ^2^ (95% CI)	*p*
**Child**		
Weight, kg	−6.7 (−13.9; 1.3)	0.094
Length, cm	−2.4 (−5.0; 0.3)	0.083
MUAC, cm	−6.5 (−13.8; 1.4)	0.106
LAZ	−5.6 (−12.4; 1.8)	0.132
WAZ	−6.0 (−13.2; 1.9)	0.130
WLZ	−3.0 (−11.6; 6.3)	0.512
Stunting	−6.7 (−20.7; 9.9)	0.408
Wasting	7.0 (−22.8; 48.3)	0.683
Underweight	3.3 (−13.5; 23.5)	0.717
Low MUAC (MUAC ≤ 12.5 cm)	32.4 (−4.8; 84.1)	0.095
Breastfeeding	19.0 (−1.5; 43.8)	0.071
Iron rich foods	−23.9 (−38.8; −5.4)	0.014
Adequate dietary diversity	−11.4 (−25.9; 6.0)	0.186
Minimum meal frequency	−10.1 (−24.5; 7.0)	0.231
CRP, mg/L	5.4 (−0.2; 11.4)	0.058
Elevated CRP (CRP > 5 mg/L)	19.6 (−7.5; 54.7)	0.172
AGP, g/L	30.3 (12.4; 51.1)	<0.001
Elevated AGP (AGP > 1 g/L)	39.2 (13.2; 70.6)	0.002
RBP, mg/dL	−14.3 (−36.5; 15.8)	0.316
Ferritin, µg/L	6.9 (−4.0; 19.1)	0.221
Low ferritin (pF < 12 µg/L)	−4.7 (−24.5; 20.3)	0.687
sTfR, mg/L	6.9 (−11.7; 29.4)	0.495
High sTfR (sTfR > 8.3 mg/L)	23.5 (4.1; 46.7)	0.016
**Maternal**		
Age	−1.0 (−2.3; 0.3)	0.135
Education	−4.3 (−18.5; 12.3)	0.589
Marital status (couple)	25.0 (−23.0; 103.0)	0.367
BMI	1.8 (−0.8; 4.4)	0.172
**Household**		
SES index	−2.9 (−6.9; 1.3)	0.175
**HFIAS**		
Food secure	Ref	
Mildly food insecure	−5.6 (−24.0; 17.3)	0.604
Moderately food insecure	0.9 (−19.4; 26.3)	0.939
Severely food insecure	−7.0 (−28.1; 20.2)	0.578

^1^ Estimates units are percentage, *n* = 512 for child characteristics; *n* = 499 for maternal and household characteristics. Linear regression models adjusted for age at baseline, sex and district were used to examine potential risk factors associated with HCC at baseline. AGP: α_1_-acid glycoprotein; BMI: body mass index; CRP: C-reactive protein; HFIAS: household food insecurity access scale [38]; LAZ, length-for-age *z*-score; MUAC: mid-upper arm circumference; RBP: retinol binding protein; SES: socio-economic status; sTfR: soluble transferrin receptor; WAZ, weight-for-age *z*-score; WLZ, weight-for-length *z*-score. ^2^ Hair cortisol concentration was log transformed for analysis and the estimates reported here are back transformed percent change. The exponential function in excel was used for the back-transformations. These are understood to be the percent change associated with a 1 unit increase in the variable indicated: a negative estimate means that the presence or an increase of 1 unit of a risk factor is associated with the estimate percent decrease of HCC and a positive estimate means that the presence or an increase of 1 unit of a risk factor is associated with the estimate percent increase of HCC.

**Table 4:** Associations between endline HCC and morbidity in the previous 3 months ^1^.

Variables	*n* (%)	Percent Change ^2^ (95% CI)	*p*
Reported at least 1 episode of diarrhea	238 (46.5)	3.1 (−10.4; 18.7)	0.666
Reported at least 1 episode of fever	382 (74.6)	5.4 (−10.3; 23.9)	0.520
Measured at least 1 episode of fever	60 (11.7)	6.7 (−14.2; 32.6)	0.560

^1^ Estimates units are percentage. Linear regression models adjusted for intervention group, age at endline, sex and district were used to explore associations between endline HCC and morbidity burden in the previous 3–4 months. ^2^ Hair cortisol concentration was log transformed for analysis and the estimates reported here are back transformed percent change. The exponential function in excel was used for the back-transformations.
